# Supplementary material for: Transcriptome and Proteome Analyses Revealed Differences in JEV-Infected PK-15 Cells in Response to Ferroptosis Agonists and Antagonists
Source: Animals (Basel). 2024 Dec 5;14(23):3516. doi: 10.3390/ani14233516 (PMC11640317; doi:10.3390/ani14233516)
Supplement: Supplementary file 1 [file animals-14-03516-s001.zip › Supplementary Figures.pdf]

Supplementary Figures

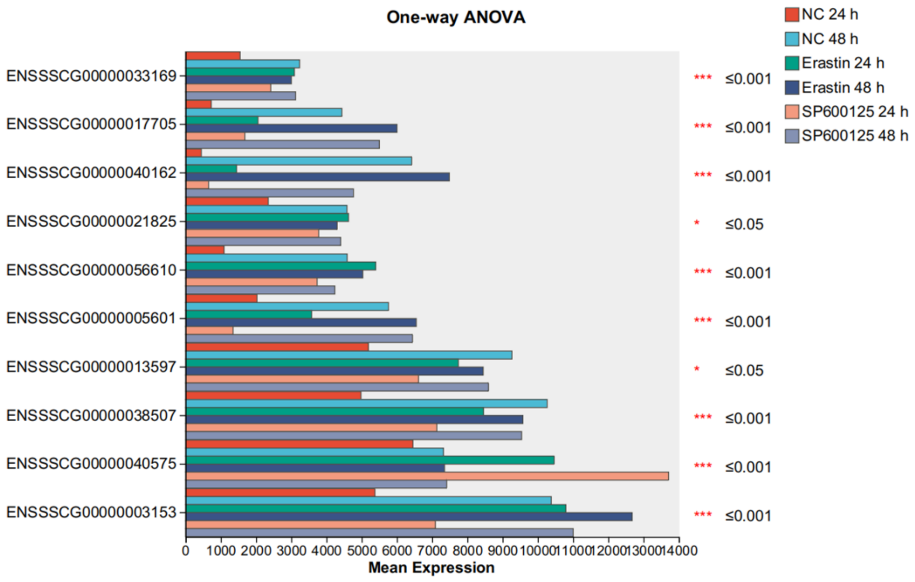

Figure S1. Multi-group difference analysis of DEGs.

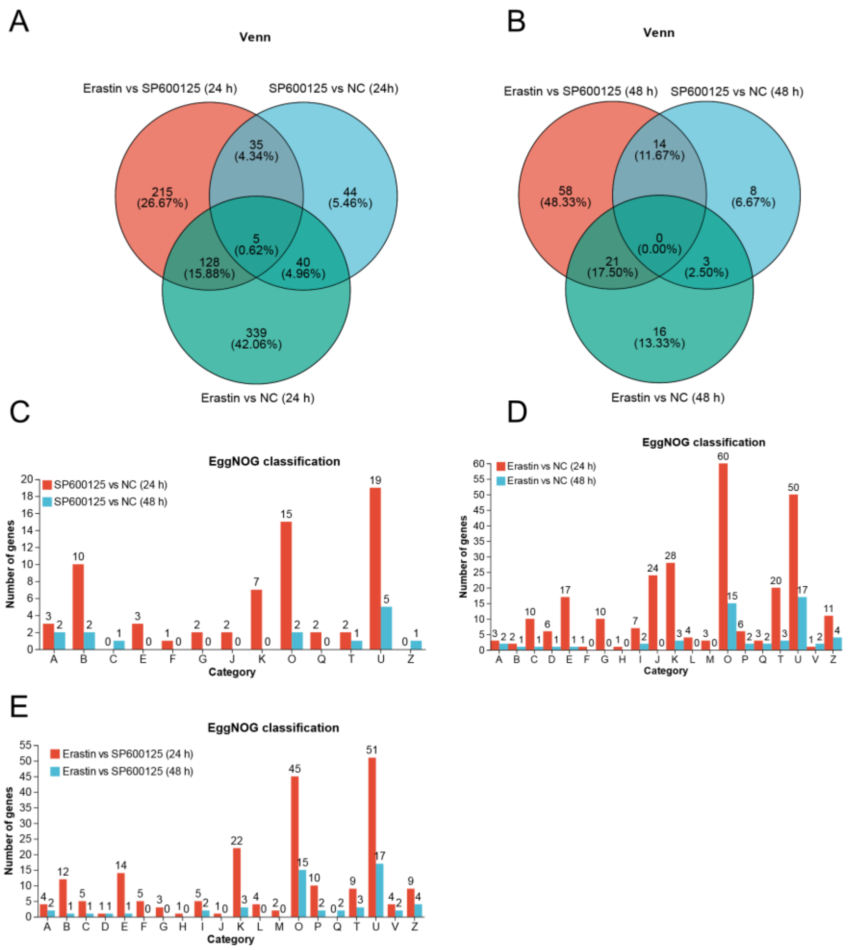

Figure S2. Venn and EggNOG analysis of DEGs:

(A) Venn of DEGs 24 h after ferroptosis agonist or inhibitor treated; (B) Venn of DEGs 48 h after ferroptosis agonist or inhibitor treated; (C) EggNOG classification of DEGs SP600125 VS NC groups; (D) EggNOG classification of DEGs Erastin VS NC groups; and (E) EggNOG classification of DEGs Erastin VS SP600125 groups.

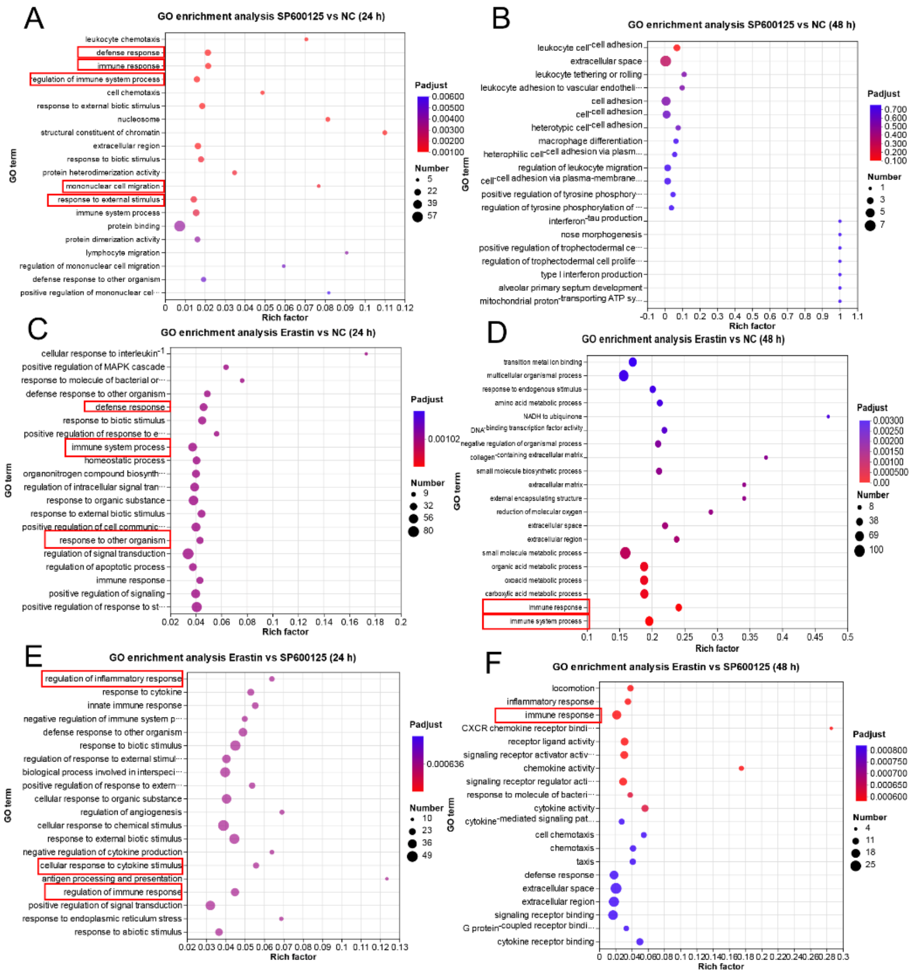

Figure S3. GO functional enrichment analysis of DEGs:
(A, B) The top 20 GO functional enrichment between SP600125 and NC groups; (C, D) The top 20 GO functional enrichment between Erastin and NC groups; and (E, F) The top 20 GO functional enrichment between Erastin and SP600125 groups.

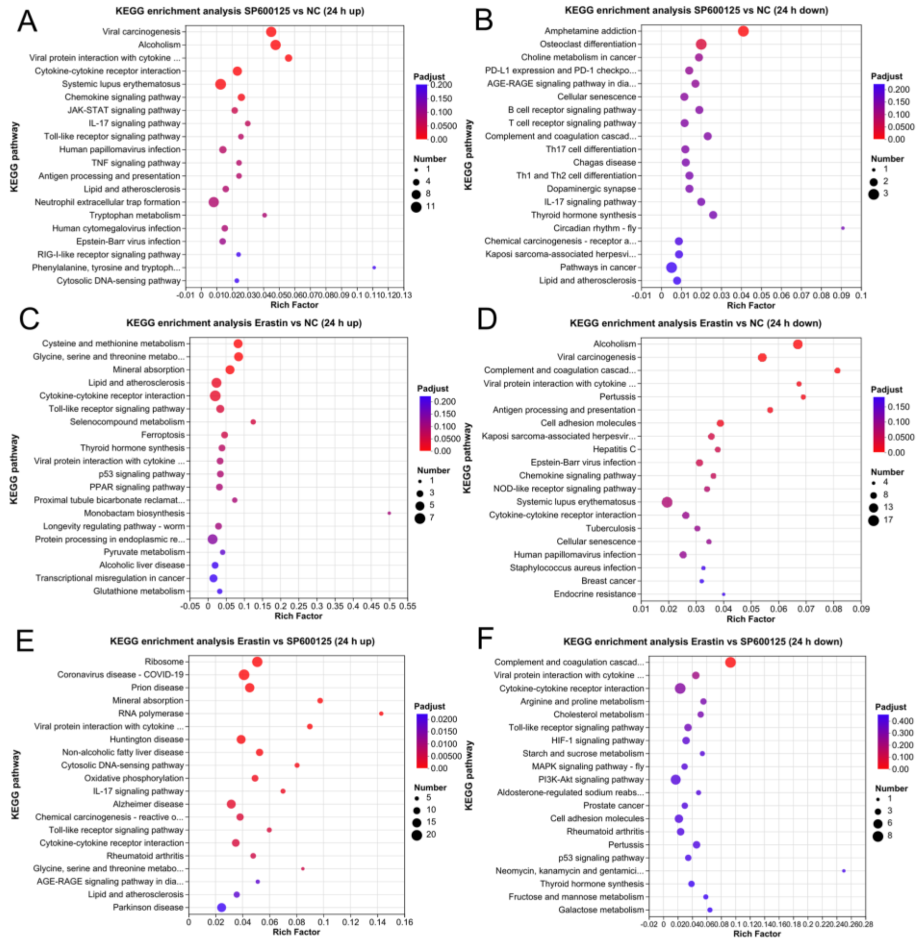

Figure S4. Pathways enrichment analysis of upregulated or downregulated DEGs: (A) The top 20 KEGG enrichment pathways of upregulated DEGs between SP600125 and NC groups; (B) The top 20 KEGG enrichment pathways of downregulated DEGs between SP600125 and NC groups; (C) The top 20 KEGG enrichment pathways of upregulated DEGs between Erastin and NC groups; (D) The top 20 KEGG enrichment pathways of downregulated DEGs between Erastin and NC groups; (E) The top 20 KEGG enrichment pathways of upregulated DEGs between Erastin and SP600125 groups; and (F) The top 20 KEGG enrichment pathways of downregulated DEGs between Erastin and SP600125 groups.

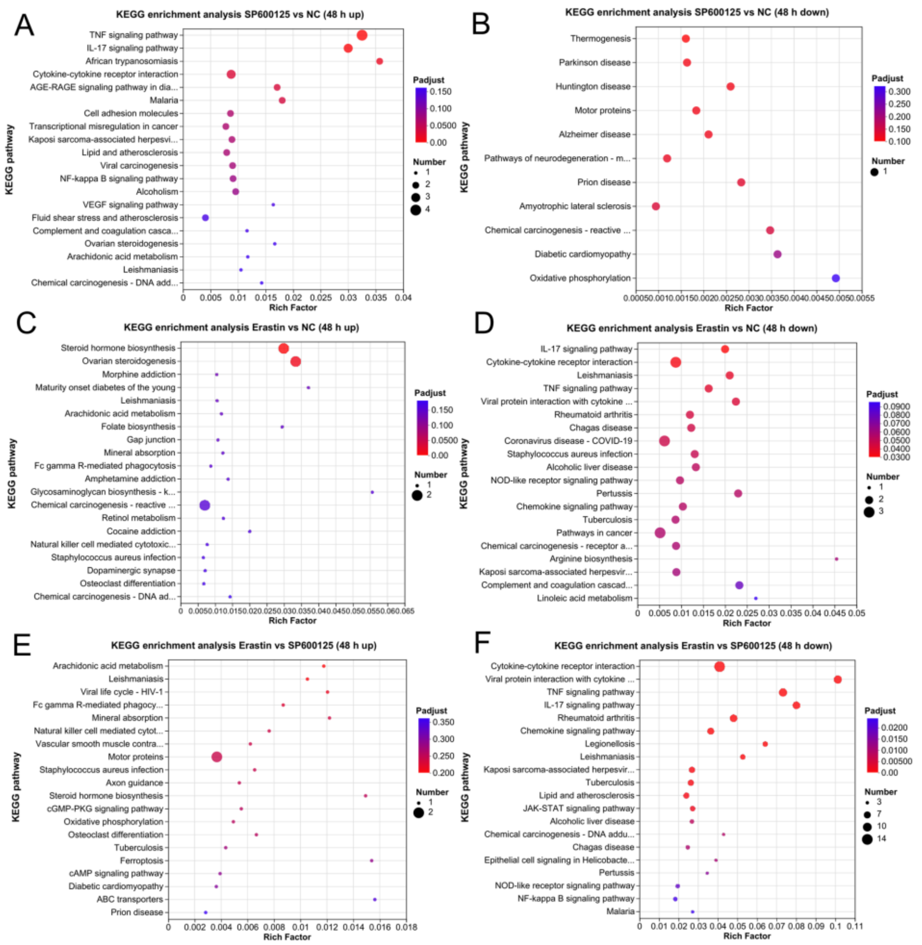

Figure S5. Pathways enrichment analysis of upregulated or downregulated DEGs: (A) The top 20 KEGG enrichment pathways of upregulated DEGs between SP600125 and NC groups; (B) The top 20 KEGG enrichment pathways of downregulated DEGs between SP600125 and NC groups; (C) The top 20 KEGG enrichment pathways of upregulated DEGs between Erastin and NC groups; (D) The top 20 KEGG enrichment pathways of downregulated DEGs between Erastin and NC groups; (E) The top 20 KEGG enrichment pathways of upregulated DEGs between Erastin and SP600125 groups; and (F) The top 20 KEGG enrichment pathways of downregulated DEGs between Erastin and SP600125 groups.

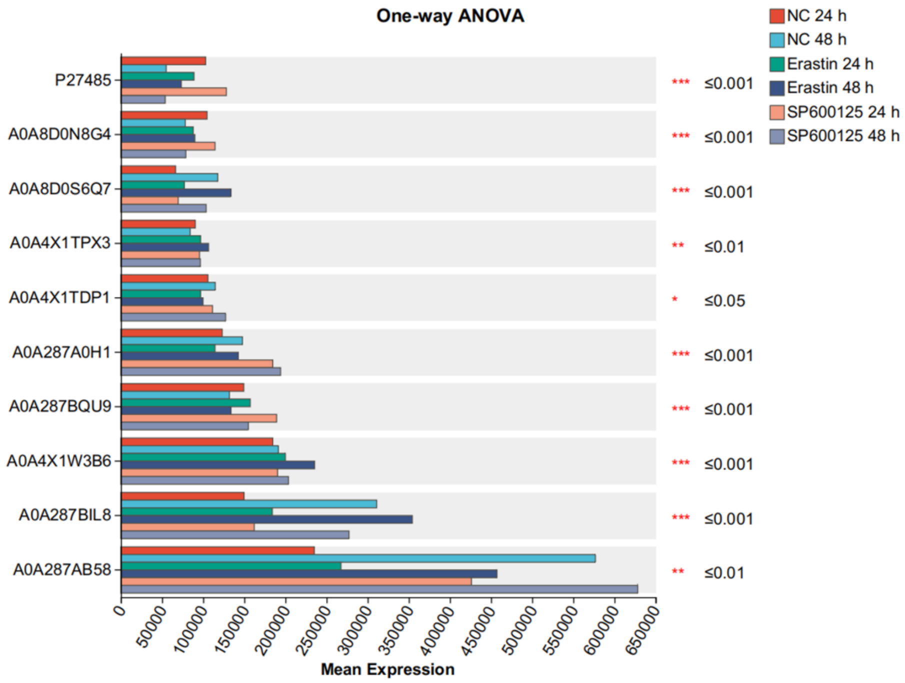

Figure S6. Multi-group difference analysis of DEPs.

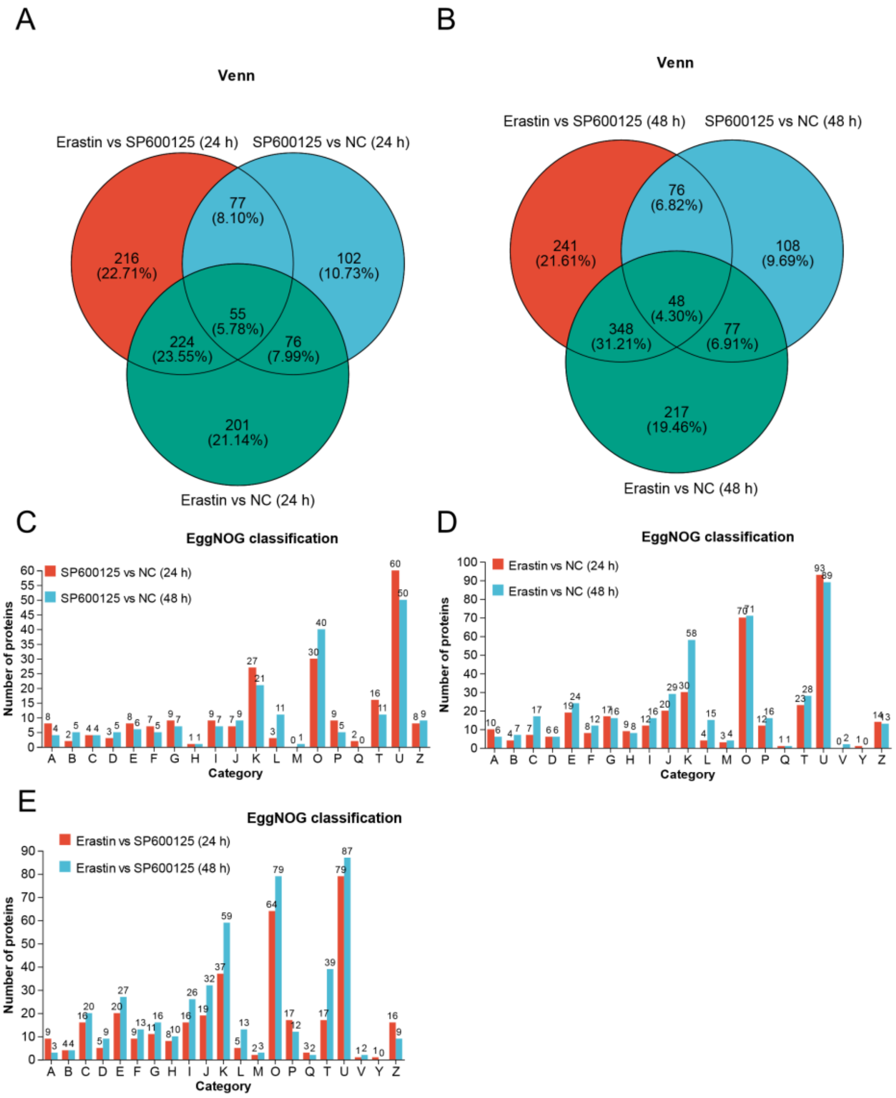

Figure S7. Venn and EggNOG analysis of DEPs:
(A) Venn of DEPs 24 h after ferroptosis agonist or inhibitor treated; (B) Venn of DEPs 48 h after ferroptosis agonist or inhibitor treated; (C) EggNOG classification of DEPs SP600125 VS NC groups; (D) EggNOG classification of DEPs Erastin VS NC groups; and (E) EggNOG classification of DEPs Erastin VS SP600125 groups.

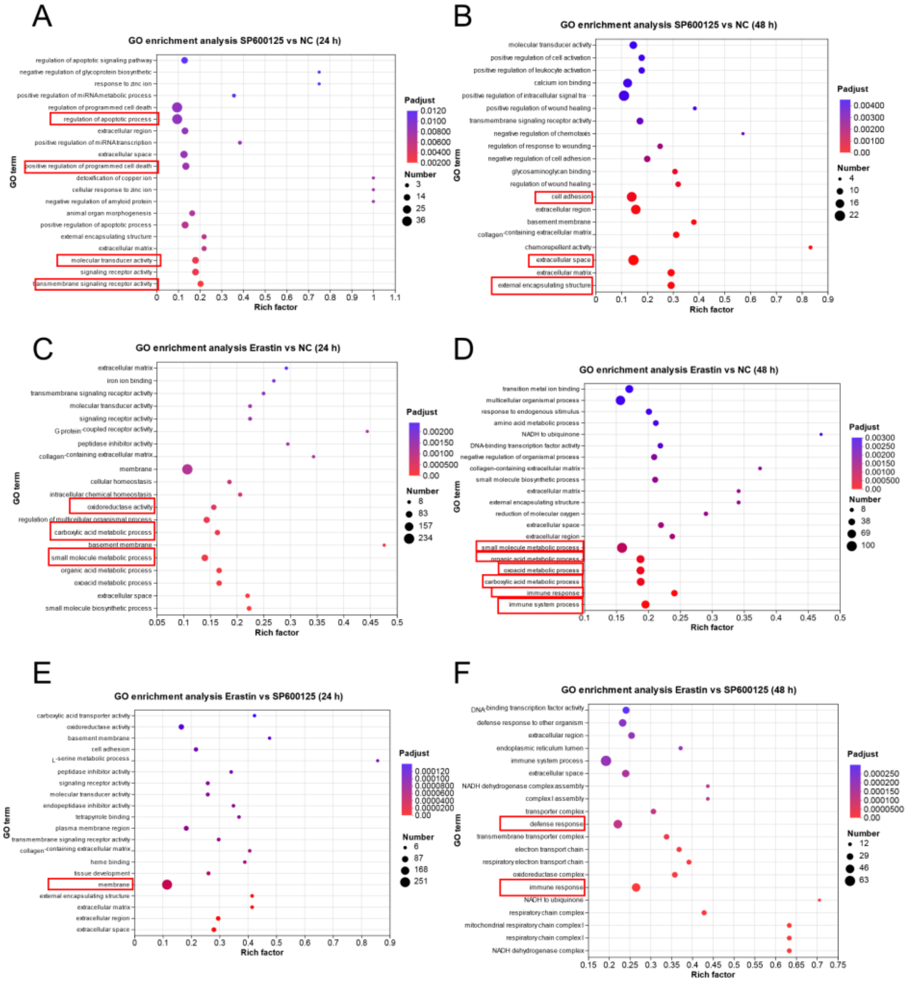

Figure S8. GO functional enrichment analysis of DEPs:
(A, B) The top 20 GO functional enrichment between SP600125 and NC groups; (C, D) The top 20 GO functional enrichment between Erastin and NC groups; and (E, F) The top 20 GO functional enrichment between Erastin and SP600125 groups.

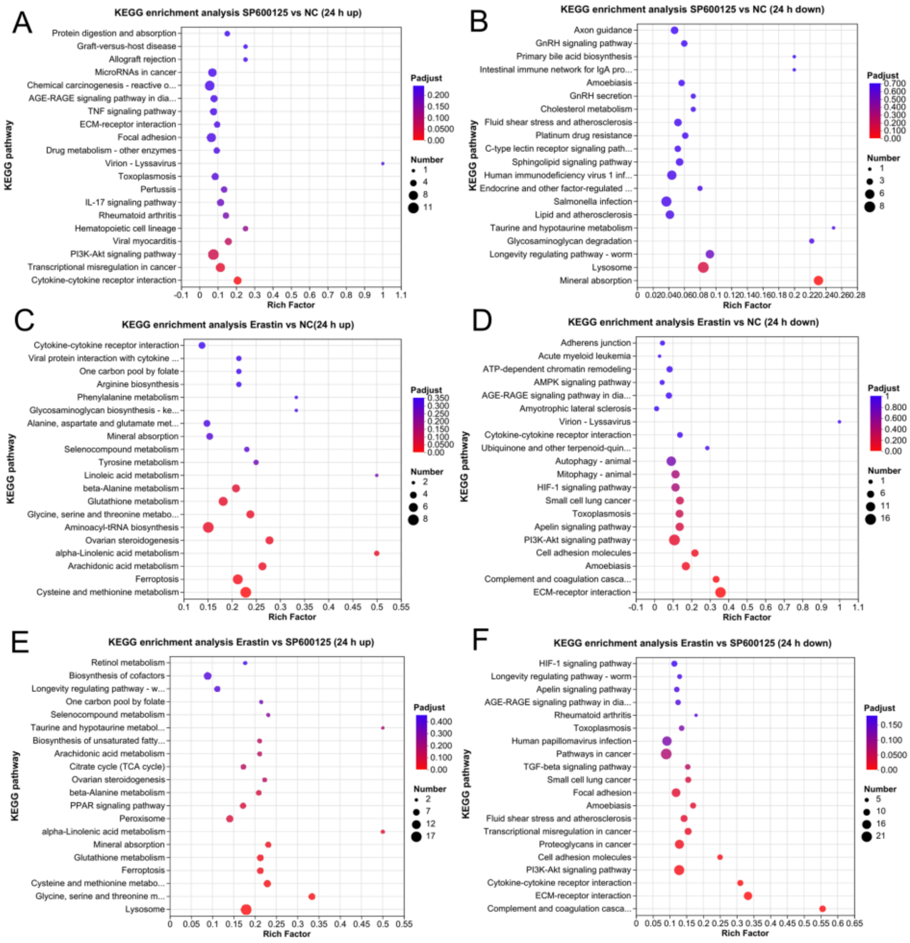

Figure S9. Pathways enrichment analysis of upregulated or downregulated DEPs: (A) The top 20 KEGG enrichment pathways of upregulated DEPs between SP600125 and NC groups; (B) The top 20 KEGG enrichment pathways of downregulated DEPs between SP600125 and NC groups; (C) The top 20 KEGG enrichment pathways of upregulated DEPs between Erastin and NC groups; (D) The top 20 KEGG enrichment pathways of downregulated DEPs between Erastin and NC groups; (E) The top 20 KEGG enrichment pathways of upregulated DEPs between Erastin and SP600125 groups; and (F) The top 20 KEGG enrichment pathways of downregulated DEPs between Erastin and SP600125 groups.

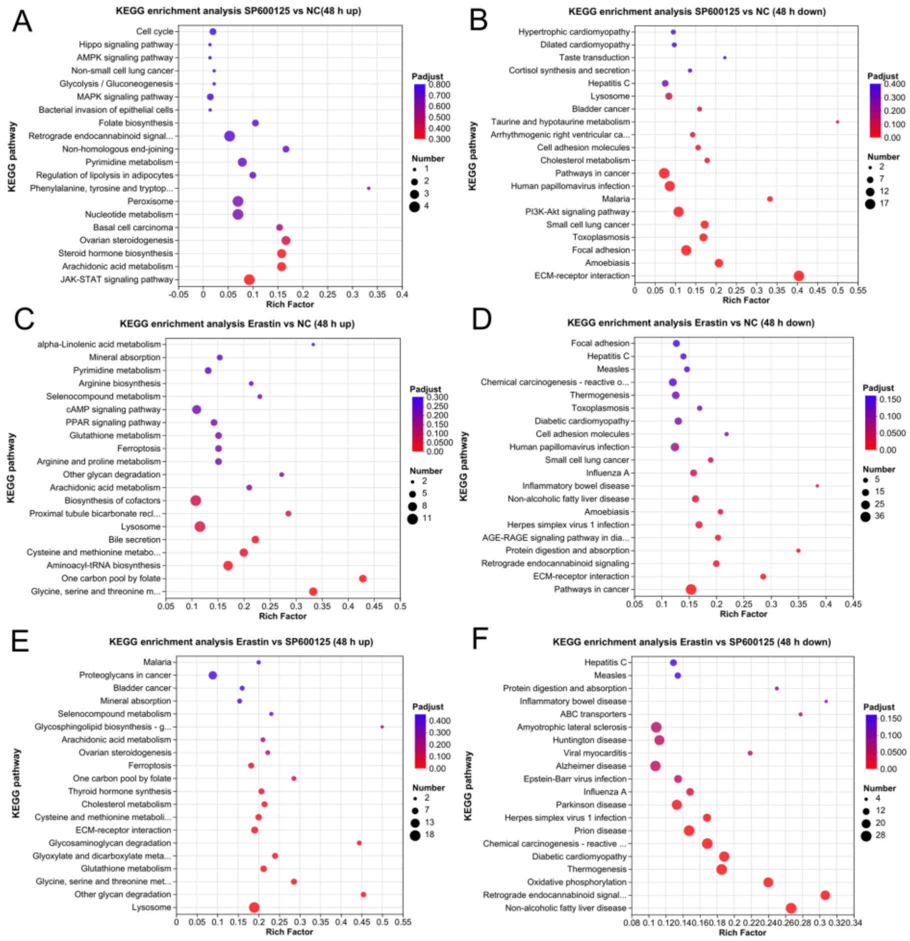

Figure S10. Pathways enrichment analysis of upregulated or downregulated DEPs: (A) The top 20 KEGG enrichment pathways of upregulated DEPs between SP600125 and NC groups; (B) The top 20 KEGG enrichment pathways of downregulated DEPs between SP600125 and NC groups; (C) The top 20 KEGG enrichment pathways of upregulated DEPs between Erastin and NC groups; (D) The top 20 KEGG enrichment pathways of downregulated DEPs between Erastin and NC groups; (E) The top 20 KEGG enrichment pathways of upregulated DEPs between Erastin and SP600125 groups; and (F) The top 20 KEGG enrichment pathways of downregulated DEPs between Erastin and SP600125 groups.

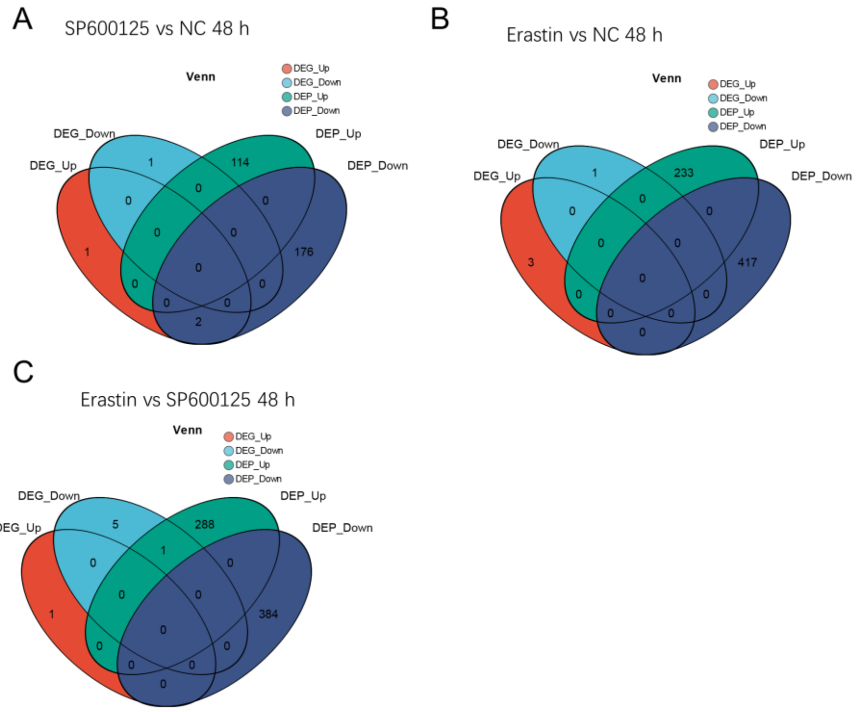

Figure S11. Venn of DEGs and DEPs:

(A) Venn of DEGs and DEPs between SP600125 or NC groups; (B) Venn of DEGs and DEPs between Erastin or NC groups; and (C) Venn of DEGs and DEPs between Erastin or SP600125 groups.

WB original images of figure 2

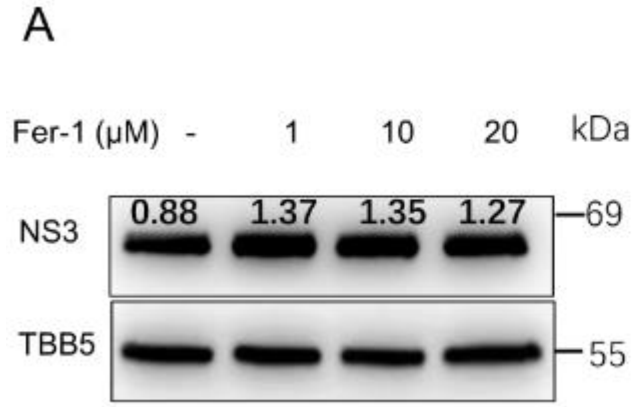

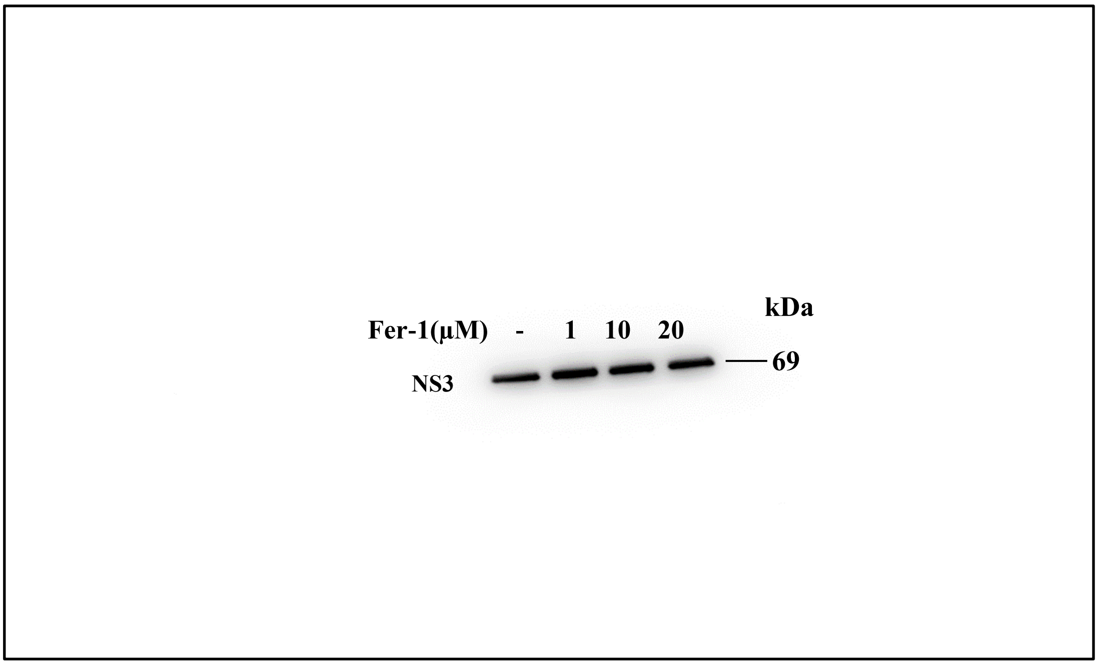

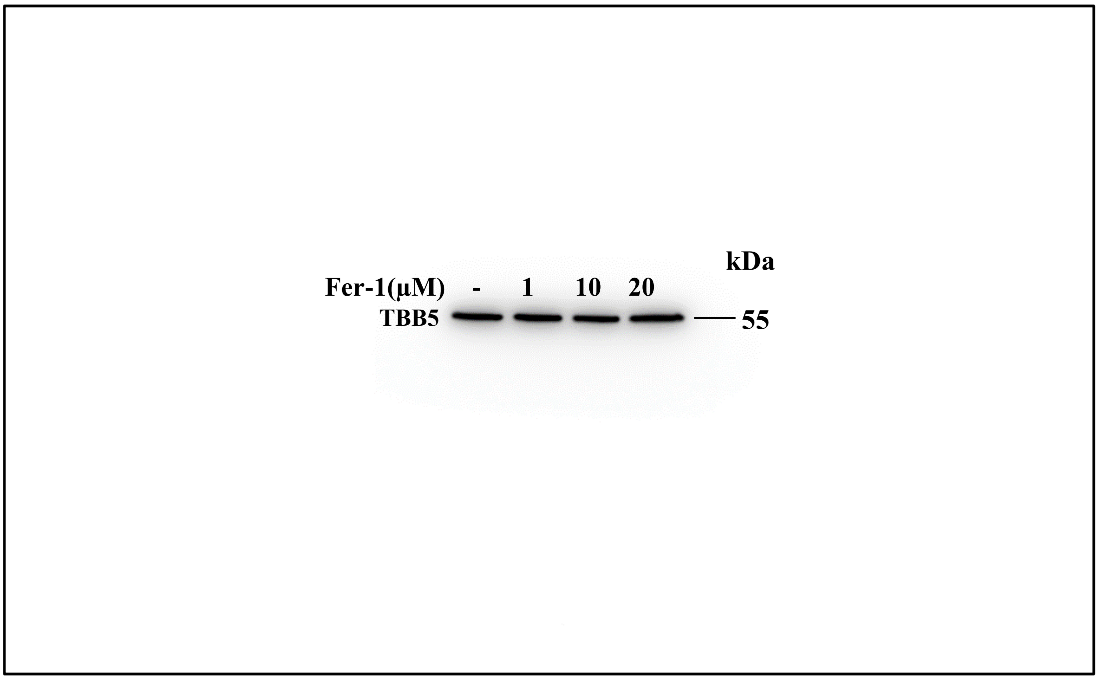

B

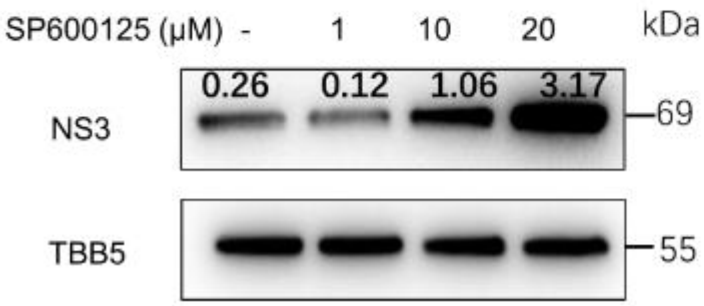
SP600125 (μM) - 1 10 20
kDa
0.26 0.12 1.06 3.17
NS3
69
TBB5
55

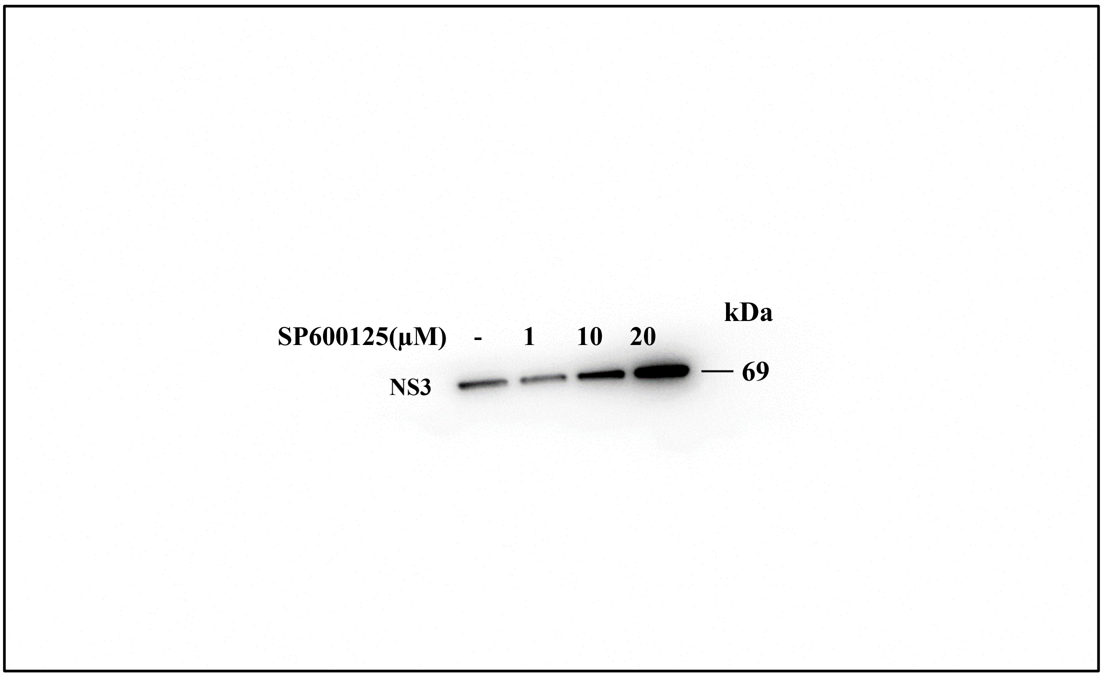
kDa
SP600125(μM) - 1 10 20
NS3
69

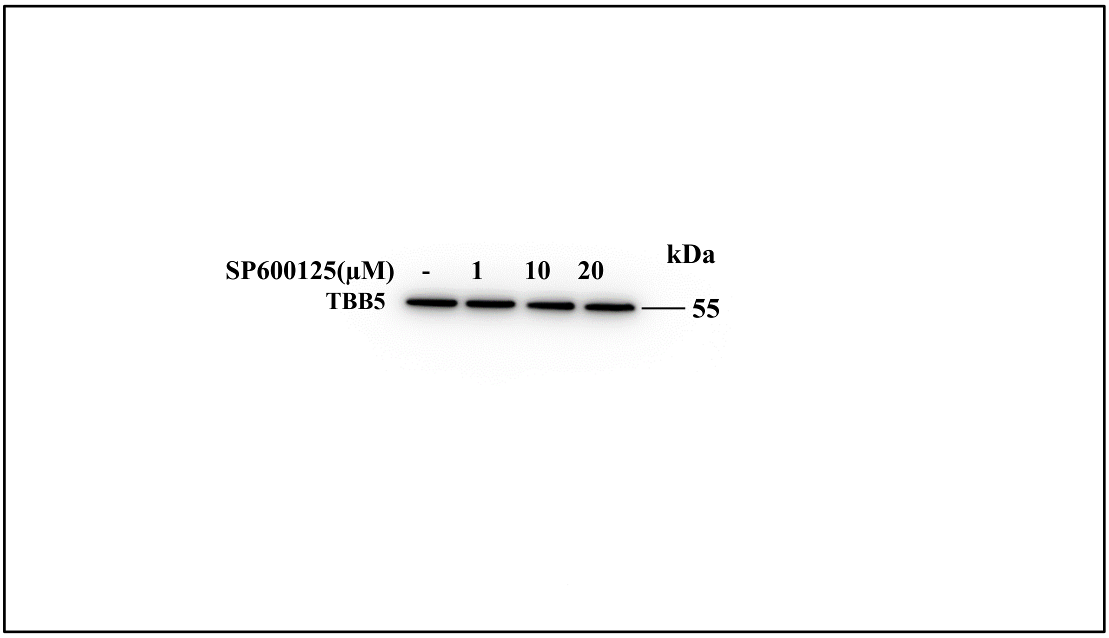
kDa
SP600125(μM) - 1 10 20
TBB5
55

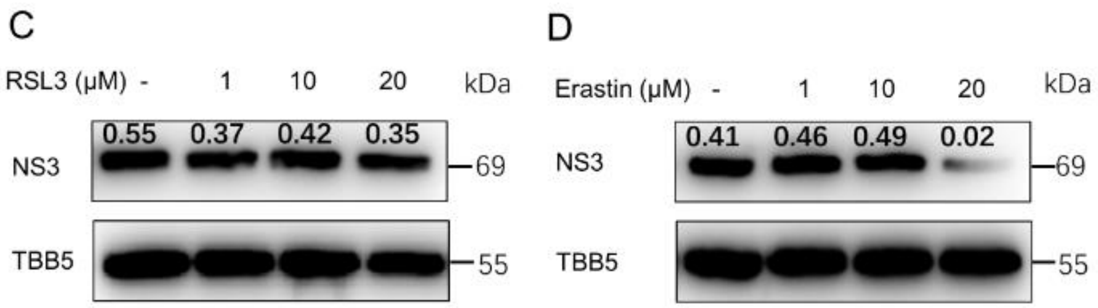
C
RSL3 (µM) - 1 10 20 kDa
NS3
0.55 0.37 0.42 0.35
69
TBB5
55
D
Erastin (µM) - 1 10 20 kDa
NS3
0.41 0.46 0.49 0.02
69
TBB5
55

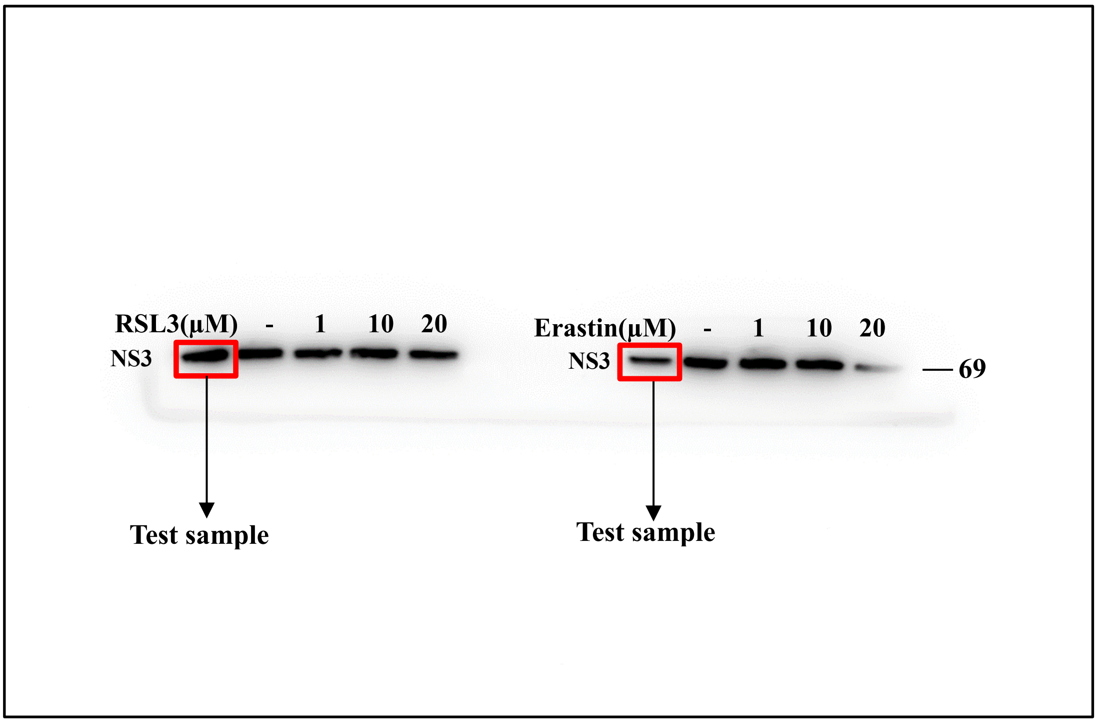
RSL3(µM) - 1 10 20
NS3
Erastin(µM) - 1 10 20
NS3
69
Test sample
Test sample

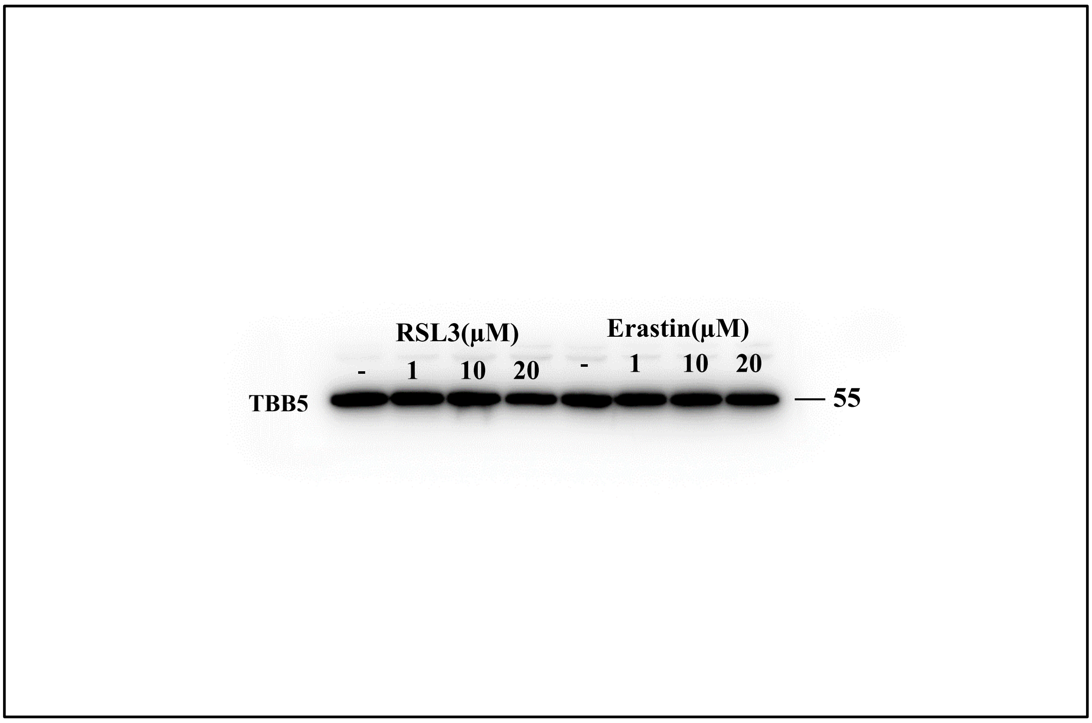
RSL3(µM)
- 1 10 20
Erastin(µM)
- 1 10 20
TBB5
55

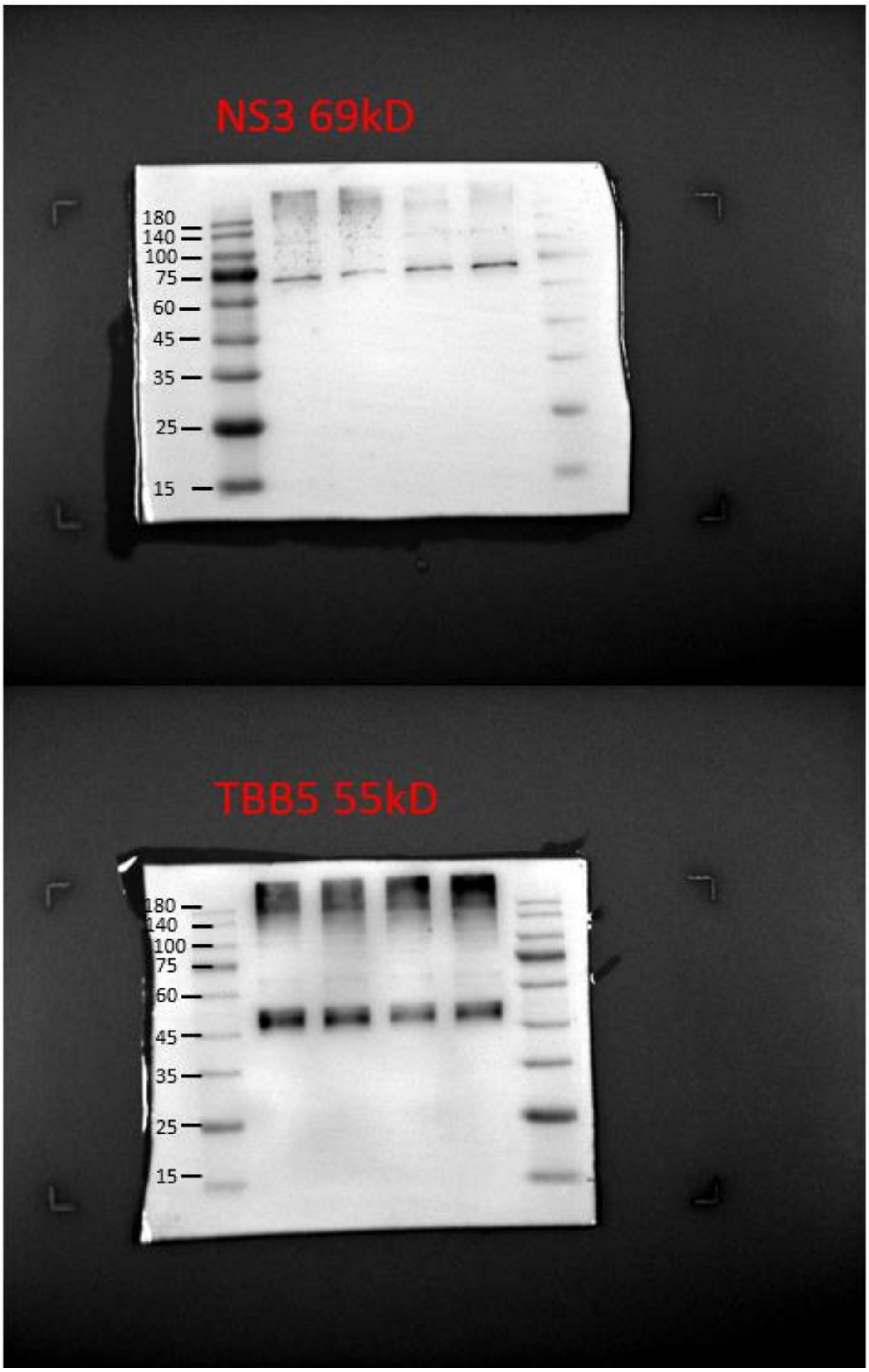
NS3 69kD
180
140
100
75
60
45
35
25
15
TBB5 55kD
180
140
100
75
60
45
35
25
15
